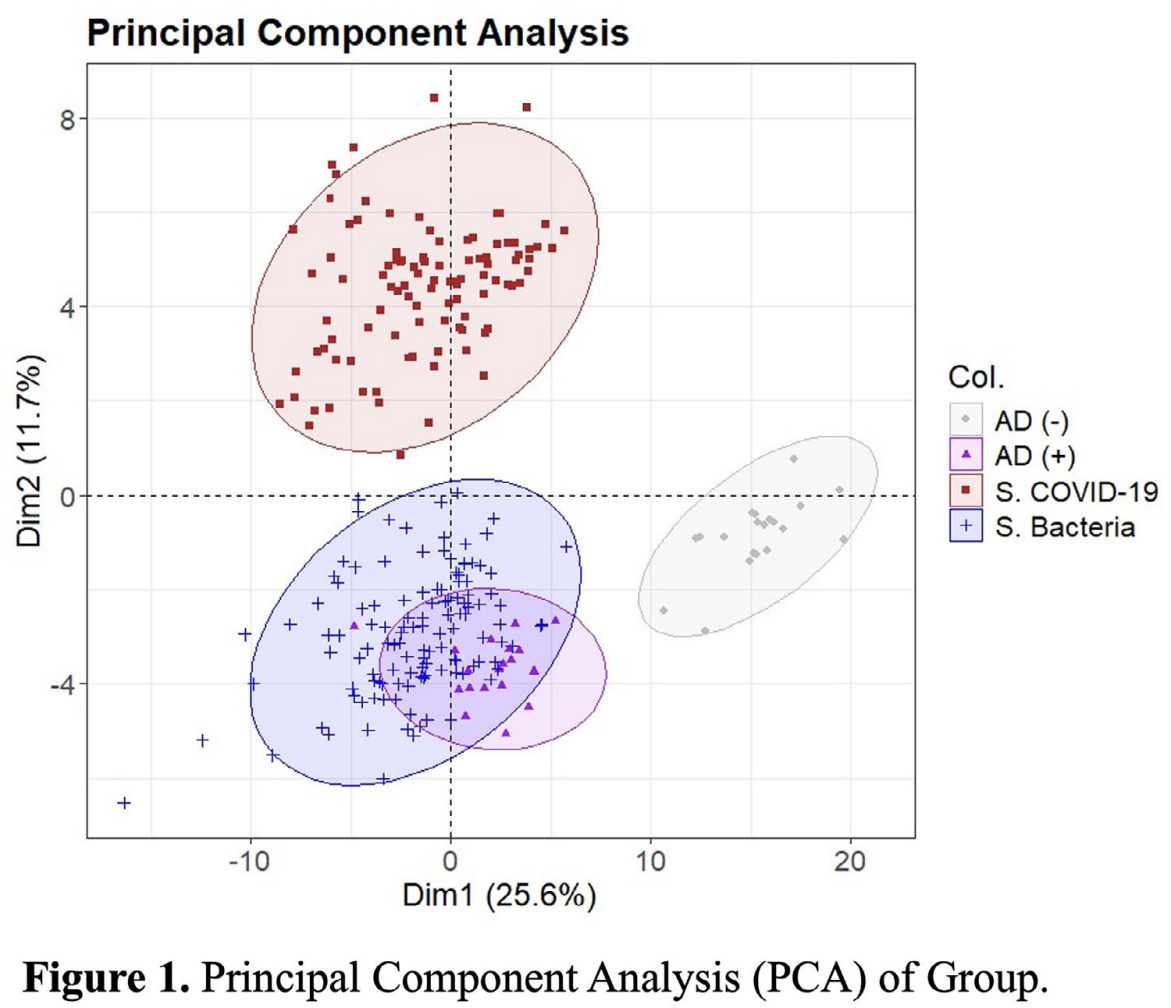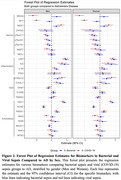# Shared Plasma Biomarker Signatures Between Sepsis and Alzheimer’s Disease

**DOI:** 10.1002/alz70861_108350

**Published:** 2025-12-23

**Authors:** Tatiana Barichello, Bruno Kluwe‐Schiavon, Giselli Scaini, Mohd Tayyab, Diogo Dominguini, Josiane Budni, Fabricia Petronilho, Cristiane Ritter, Rodrigo Morales, Fernando A. Bozza, Mervyn Singer, Felipe Dal‐Pizzol

**Affiliations:** ^1^ McGovern Medical School, University of Texas Health Science Center at Houston, Houston, TX USA; ^2^ University of Southern Santa Catarina, Criciuma, SC Brazil; ^3^ The University of Texas Health Science Center at Houston, Houston, TX USA; ^4^ Hospital São José Research Center, Criciuma, SC Brazil; ^5^ Oswaldo Cruz Foundation, Rio de Janeiro, RJ Brazil; ^6^ University College London, London, United Kingdom UK

## Abstract

**Background:**

Clinical studies indicate an association between hospital‐diagnosed infections and the development and progression of AD and other types of dementia. However, the mechanisms by which infection contributes to the decline in cognitive function and the worsening of AD are unknown. This study investigated plasma proteomic biomarkers in patients with sepsis—a life‐threatening condition characterized by organ dysfunction resulting from a dysregulated host response to infection—admitted to intensive care units (ICUs). We evaluated their overlap with biomarkers associated with AD pathology.

**Methods:**

The study included 263 participants: 122 with bacterial sepsis, 101 with viral sepsis (COVID‐19), 20 with AD, and 20 healthy controls (HCs). AD patients were significantly older (median age 77.0 years, IQR: 73.5–79.0) compared to the sepsis cohorts (bacterial: 68.0 [53.0–79.8], viral: 67.0 [57.0–73.0], *p* < 0.001). The proportion of APOE carriers was highest in the AD group (80%), compared to bacterial (19.7%) and viral sepsis (23.8%) patients (p < 0.001). Illness severity scores (SOFA and SAPS3) were significantly higher in bacterial sepsis patients compared to those with viral sepsis (p < 0.01). 120 CNS proteomic plasma biomarkers of glial cells, neuroinflammation, neurodegeneration, and blood‐brain barrier integrity were measured by a proteomic liquid biopsy platform with attomolar sensitivity and high multiplexing NULISA™.

**Results:**

Principal component analysis (PCA) revealed that bacterial sepsis patients exhibited partial overlap with the AD‐positive group, suggesting shared biomarker patterns. In contrast, the viral sepsis group formed a distinct cluster with minimal overlap, and AD‐negative controls remained well separated from all other groups. Furthermore, bacterial sepsis showed several biomarkers shared by patients with AD. This includes neurodegenerative and neurological proteins such as neurogranin (NRGN), neuropeptide Y (NPY), synaptosomal‐associated protein 25 (SNAP25), microtubule‐associated protein tau (MAPT), alpha‐synuclein (SNCA), amyloid‐beta isoforms (Aβ_1‐42_, Aβ_1‐40_, Aβ_1‐38_), phosphorylated tau species (pTau181, pTau217, pTau231), and phosphorylated TDP‐43 (pTDP43.400), all of which are implicated in AD pathology.

**Conclusion:**

Our results indicate that sepsis and AD share common biomarkers, suggesting that sepsis may exacerbate AD progression and that both conditions may have overlapping pathophysiological mechanisms. Understanding these similarities could be crucial for developing novel therapies targeting neuroinflammation, neurodegeneration, and PICs.